# Prevalence of intestinal parasites, with emphasis on the molecular epidemiology of *Giardia duodenalis* and *Blastocystis* sp., in the Paranaguá Bay, Brazil: a community survey

**DOI:** 10.1186/s13071-018-3054-7

**Published:** 2018-08-30

**Authors:** Raimundo Seguí, Carla Muñoz-Antoli, Debora R. Klisiowicz, Camila Y. Oishi, Pamela C. Köster, Aida de Lucio, Marta Hernández-de-Mingo, Paula Puente, Rafael Toledo, José G. Esteban, David Carmena

**Affiliations:** 1 Department of Pharmacy, Pharmaceutical Technology and Parasitology, Parasitology Area, Faculty of Pharmacy, Valencia University, Avda. Vicent Andrés Estellés s/n, 46100 Burjassot, Valencia Spain; 2 Department of Basic Pathology, Biological Sciences Area, Paraná Federal University, Av. Cel. Francisco H. dos Santos 100, Curitiba, 19031 Brazil; 3 Parasitology Reference and Research Laboratory, National Centre for Microbiology, Ctra. Majadahonda-Pozuelo Km 2, 28220 Majadahonda, Madrid, Spain

**Keywords:** Intestinal parasites, Soil-transmitted helminths, Helminth, Nematode, Protozoa, Human, Community, Microscopy, Genotyping, Brazil

## Abstract

**Background:**

Intestinal protozoan parasites are major contributors to the global burden of gastrointestinal disease causing significant socioeconomic consequences. Children living in resource-poor settings with restricted access to water and sanitary services are particularly at risk of these infections.

**Methods:**

A prospective, community-based, cross-sectional survey was conducted in Paraná (southern Brazil) between May 2015 and May 2016. A total of 766 stool samples were individually collected from volunteers (male/female ratio: 0.99; age range: 0–76 years) and used for investigating the presence of intestinal helminth and protozoan species by routine microscopic procedures including the Kato-Katz and modified Ritchie concentration methods and the Ziehl-Neelsen stain technique. Quantitative real-time PCR confirmed microscopy-positive samples for *Giardia duodenalis* and the assemblages and sub-assemblages determined by multilocus sequence-based genotyping of the glutamate dehydrogenase (*gdh*) and β-giardin (*bg*) genes of the parasite. Identification of *Blastocystis* subtypes was carried out by amplification and sequencing of a partial fragment of the small-subunit ribosomal RNA gene (*SSU* rDNA) of this heterokont microorganism.

**Results:**

Overall, 46.1% (353/766) of the participants were infected/colonised by at least one intestinal parasite/commensal species. Protozoan and helminth species were detected in 42.7% and 10.1% of the surveyed population, respectively. *Blastocystis* sp. (28.2%), *Endolimax nana* (14.9%), and *Giardia duodenalis* (11.0%) were the most prevalent species found among protozoans and *Ascaris lumbricoides* (5.0%), *Trichuris trichiura* (4.6%) and hookworms (1.0%) among helminths. A total of 38 *G. duodenalis*-positive samples were genotyped at *gdh* and *bg* markers, revealing the presence of the sub-assemblages AII (47.4%), AII/AIII (2.6%), BIII (5.3%), BIV (26.3%) and BIII/BIV (13.1%). Two samples (5.3%) were only identified as assemblage B. AII was predominantly found in females aged 5–9 years and was associated with a higher likelihood of reporting gastrointestinal symptoms. A total of 102 *Blastocystis*-positive samples were successfully subtyped at the *SSU* rRNA gene revealing the presence of ST1 (36.3%), ST2 (15.7%), ST3 (41.2%), ST4 (2.9%), ST6 (1.0%) and ST8 (2.9%).

**Conclusions:**

Data presented here indicate that enteric parasites still represent a pressing health concern in Paraná, Brazil, probably due to sub-optimal water, sanitation and hygiene conditions. A mostly anthroponotic origin is suspected for *G. duodenalis* and *Blastocystis* sp. infections.

**Electronic supplementary material:**

The online version of this article (10.1186/s13071-018-3054-7) contains supplementary material, which is available to authorized users.

## Background

A wide range of helminth and protozoan species can infect or colonise the gastrointestinal tract of humans and animals. These organisms are typically transmitted through the faecal-oral route indirectly by ingestion of contaminated food, water, soil, or fomites. Direct transmission *via* person-to-person or animal-to-person contact is also possible for several species [1]. Consequently, the occurrence of enteric parasites (EPs) is strongly linked to poverty, lack of or insufficient access to safe drinking water, inadequate sanitation and poor hygiene practices and education [2]. Not surprisingly, EPs disproportionally affect children living in low- and middle-income settings in developing countries [3–5], although these infections are also important contributors to the disease burden in the developed world [6].

EPs of public health concern include the protozoa *Cryptosporidium* spp., *Entamoeba histolytica* and *Giardia duodenalis*. *Cryptosporidium* spp. are the second biggest cause, after rotavirus, of diarrheal death in children under five years in sub-Saharan Africa [7], whereas invasive amoebic infection by *E. histolytica* affects 50 million people worldwide each year, resulting in 40,000–100,000 deaths annually [8]. Although rarely fatal, more than 200 million people are estimated to have symptomatic giardiasis by *G. duodenalis* only in developing countries [9], most of them caused by the zoonotic assemblages A and B of the parasite [10]. Other EPs such as the heterokont *Blastocystis* sp. and the protozoan *Dientamoeba fragilis* appear much more common than previously thought, especially in healthy individuals [11–13] and have been associated with gastrointestinal disorders including irritable bowel syndrome [14]. Because of its high degree of genetic diversity, a total of 17 subtypes (STs) with marked differences in host specificity and geographical distribution have been recognised within *Blastocystis*, with STs 1–4 accounting for ~90% of the human infections reported globally [15].

The most common helminthic EPs in humans are the soil-transmitted nematodes *Ascaris lumbricoides*, *Trichuris trichiura*, *Ancylostoma duodenale*, *Necator americanus* and *Strongyloides stercoralis*, and cestodes of the family Taeniidae, namely *Taenia saginata* and *T. solium*. Infections by these species are part of the 17 neglected tropical diseases listed and prioritised by the World Health Organization [16]. Soil-transmitted helminths (STHs) affect almost one-sixth of the global population and result in a broad spectrum of asymptomatic to symptomatic clinical manifestations, including intestinal bleeding, loss of appetite, diarrhoea, small bowel obstruction, and reduced absorption of micronutrients [5, 17]. Human taeniosis typically causes few or no symptoms, although *T. solium* cysticerci can establish in the central nervous system causing neurocysticercosis associated with some neurological disorders and even deaths [18]. Aside from cryptosporidiosis and invasive amoebiasis, EPs are considered debilitating rather than lethal infections with a significant socioeconomic and public health impact [17, 19]. Because of their insidious effects on the health and nutritional status of the host, infections by EPs in children have been proven to cause stunting and chronic anaemia, leading to cognitive impairment and failure to thrive and hindering socioeconomic development [17, 20–26].

The current epidemiology and clinical significance of EPs in the Paranaguá Bay (Paraná State, south Brazil) are poorly understood due to insufficient detection and surveillance data at the community level. In an early study conducted more than 50 years ago in the city of Paranaguá, most of the investigated population (99.3%) was infected with at least one species of EP, with STHs reaching prevalence rates ranging from 50.3% for *S. stercoralis* to 93.3% for *T. trichiura*. Among the protozoa, *Entamoeba coli* was the species more commonly identified [27]. In a subsequent study carried out in 2002, the overall infection rate for EPs decreased to 78.8%, with *A. lumbricoides* (52.9%) and *G. duodenalis* (10.6%) being identified as the most prevalent helminth and protozoan species, respectively [28]. Since then, no further epidemiological studies have been conducted in the area. Here, we present updated epidemiological data on the occurrence and geographical distribution of EPs in traditional communities and urban populations living in the Paranaguá Bay, Brazil. Additionally, and for the first time in the region, a detailed account of the genetic diversity of *G. duodenalis* and *Blastocystis* sp. is given, information extremely useful to elucidate the main transmission routes of these pathogens.

## Methods

### Study design

**Fig. 1:**
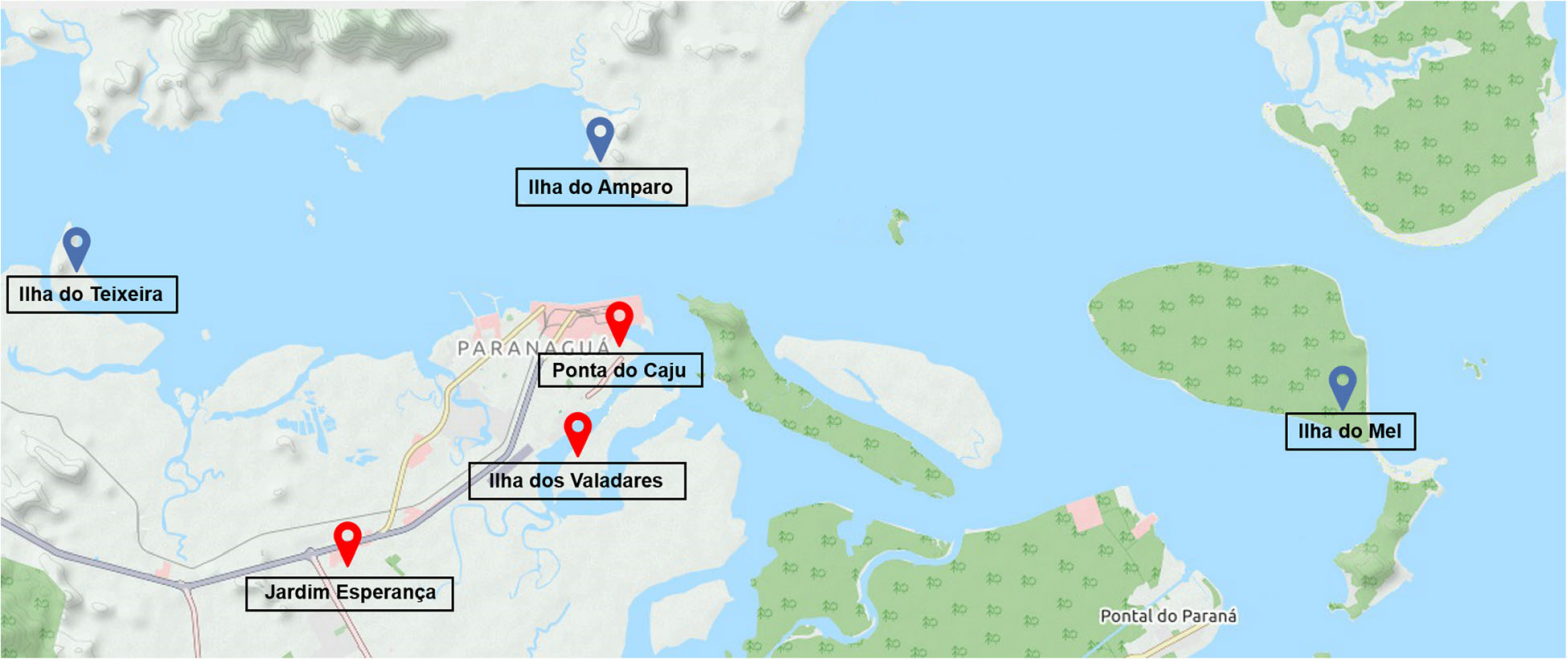
Map of the Paraná Bay, Brazil, showing the human communities sampled in the present survey. Insular or poorly accessible settings were indicated with blue labels. Urban and peri-urban settings are indicated with red labels. Image credit to www.openstreetmap.org OpenStreetMap® is open data, licensed under the Open Data Commons Open Database License by the OpenStreetMap Foundation; the cartography and documentation are licensed under the Creative Commons Attribution-ShareAlike 2.0 license

Paranaguá Bay is a large subtropical estuarine system on the coast of the Paraná State in southern Brazil (48°25'W, 25°30'S) some 110 km to the north of the capital, Curitiba. The largest town in the region is Paranaguá, with an estimated population of more than 148,000 inhabitants and a total area of 826.674 km^2^ [29]. The economy of the region is based on agriculture and trade, with a large fraction of the population living in traditional agricultural and fishing communities. During May 2015 preliminary meetings were held with regional health authorities to obtain updated census figures in the Paranaguá Bay and design the sampling campaign. A diversified sample was selected that represented the natural and socioeconomic environments of the previously mentioned population. Thus, just over 25% of the recruited volunteers came from isolated, mainly insular, communities (Ilha do Mel, Ilha do Teixeira, and Ilha do Amparo) in which motor vehicles were prohibited and access was only possible by sea. The remaining 75% were inhabitants from urban and peri-urban areas including the neighbourhoods of Jardim Esperança, and Ponta do Cajú and the island of Ilha dos Valadares (Fig. 1). A prospective cross-section epidemiological study was conducted between May 2015 and May 2016. The sample size was estimated at least on 1000 individuals of all age groups based on a ≥ 20% intestinal parasites prevalence previously reported in Brazil [30, 31], a marginal error of 2.5%, and a non-response rate of 35%.

### Sampling

A series of sensitisation meetings were held with local community leaders, school principals, and parent's representatives to provide information on the goals, procedures involved, and potential benefits of participating in this research project, and to seek for collaboration and logistic support. Sampling kits included a sterile polystyrene flask, instructions on how to take a stool sample safely, and a standardised epidemiological questionnaire covering socio-demographic (age, gender, the area of residence) and clinical (symptoms at the moment of sampling, stool consistency) data. Family households in insular areas were individually visited once and pre-labelled sampling kits distributed among all family members. Epidemiological questionnaires were filled and completed at the time of the visit, whereas stool samples were collected in the following days through local healthcare facilities or community health workers. In urban and peri-urban areas a total of three primary schools were visited, and consenting participants were provided with pre-labelled sampling kits. Collection of stool samples and epidemiological questionnaires were organised in collaboration with the schools at scheduled times and reviewed for matching and completeness. In all cases, a single stool sample was obtained per participant.

### Stool sample processing

Stool samples were immediately transported to the Department of Basic Pathology, Biological Sciences Area, Paraná Federal University (Curitiba, Brazil), and processed within 12 h of collection. Following the diagnostic algorithm recommended by the World Health Organization [32], a small amount of fresh, unpreserved faecal sample was analysed by the Kato-Katz method to estimate the burden and egg excretion of *Schistosoma mansoni* and soil-transmitted helminths in stool samples [33]. The test was conducted using a commercially available assay (Helm test, Fiocruz, Brazil) according to the manufacturer's specifications. A second aliquot was preserved in a vial containing 70% ethanol intended explicitly for extracting and purifying genomic DNA. This sub-sample set was shipped to the University of Valencia, Valencia, Spain, for downstream molecular analyses. Finally, the remaining material was mixed with an adequate amount of normal saline, filtered through sterile commercial gauze to remove debris and homogenise the faecal material, and fixed with 10% formalin in a 1:3 proportion for coproparasitological examination by the modified Ritchie concentration technique (MRCT). This method is particularly suited for improving the detection of intestinal parasites in stool specimens [34]. For the detection of *Cryptosporidium* spp. and other coccidian protozoa, faecal smears from concentrates obtained by MRCT were produced and stained with the Modified Ziehl-Neelsen method [35]. In all cases, microscopic examination was conducted at 400× and 1000× magnification under an optical microscope Nikon SE (Tokyo, Japan). A sample was considered negative when no parasite structures (eggs, cysts, oocysts, trophozoites and larvae) were observed. In samples that tested positive for protozoan enteropathogens, morphometric measurements of (oo) cysts were carefully recorded using a calibrated ocular micrometre. The number of (oo) cysts detected were totalled and used to produce estimations of protozoan load as described in [36] with minor modifications. Briefly, a total of five fields per slide were examined and the infection loads categorised as follows: low (one to four parasite structures per slide); moderate (one parasite structure per field); and heavy (more than one parasite structure per field). After completion of diagnostic procedures, prevalence and mean infection burdens were calculated for each parasitic species found.

### DNA extraction and purification

Total DNA was extracted from ~200 mg of concentrated faecal material using the QIAamp® DNA Stool Mini Kit (Qiagen, Hilden, Germany) following the manufacturer’s instructions. Purified DNA samples (200 μl) were stored at -20 °C until use. A water extraction control was routinely included in each sample batch processed.

### Molecular detection and characterisation of *Giardia duodenalis*

Samples with a positive result for *G. duodenalis* at microscopy were re-analysed by a quantitative real-time PCR (qPCR) assay to specifically amplify a 62-bp region of the small-subunit ribosomal RNA (*SSU* rRNA) gene of the parasite [37]. PCR reactions were prepared in a final volume of 25 μl containing 3 μl of genomic DNA, 0.5 μM of the primer set Gd-80F and Gd-127R, 0.4 μM of the probe (Additional file 1: Table S1) and 12.5 μl *Taq*Man® Gene Expression Master Mix (Applied Biosystems, CA, USA). The amplification protocol included an initial hold step of 2 min at 55 °C and 15 min at 95 °C followed by 45 cycles of 15 s at 95 °C and 1 min at 60 °C and was carried out on a Corbett Rotor Gene™ 6000 real-time PCR system (Qiagen). Water (no-template) and genomic DNA (positive) controls were routinely included in each PCR run.

All samples with a qPCR-positive result were subsequently assessed at the *G. duodenalis* glutamate dehydrogenase (*gdh*) and ß-giardin (*bg*) genes. A semi-nested-PCR protocol targeting a ~432-bp fragment of *gdh* was performed as described in [38]. PCR reactions were carried out in a final volume of 25 μl including 5 μl of genomic DNA and 0.5 μM of the primer pairs GDHeF/GDHiR in the primary reaction and GDHiF/GDHiR in the secondary reaction (Additional file 1: Table S1). Cycling conditions were 3 min at 95 °C (initial denaturation step) followed by 35 cycles of 95 °C for 30 s, 55 °C for 30 s and 72 °C for 1 min, with a final extension of 72 °C for 7 min. A nested-PCR protocol was used to amplify a ~511-bp fragment of the *bg* gene of *G. duodenalis* [39]. PCR reactions were conducted in a final volume of 25 μl consisting of 3 μl of genomic DNA and 0.4 μM of the primers pairs G7_F/G759_R in the primary reaction and G99_F/G609_R in the secondary reaction (Additional file 1: Table S1). Cycling parameters for the primary PCR reaction were an initial step of 95 °C for 7 min, followed by 35 cycles of 95 °C for 30 s, 65 °C for 30 s, and 72 °C for 1 min with a final extension of 72 °C for 7 min. The same conditions were used in the secondary PCR except that the annealing temperature was 55 °C.

### Molecular detection and characterisation of *Blastocystis* sp. isolates

Samples with a positive result for *Blastocystis* sp. at microscopy were re-assessed by a direct PCR method to specifically amplify a ~600-bp fragment of the *SSU* rRNA gene of the parasite [40]. Amplification reactions were carried out in a final volume of 25 μl including 5 μl of genomic DNA and 0.5 μM of the pan-*Blastocystis*, barcode primer set RD5/BhRDr (Additional file 1: Table S1). Cycling conditions consisted of one step of 95 °C for 3 min, followed by 30 cycles of 1 min each at 94, 59 and 72 °C, with an additional 2 min final extension at 72 °C.

All PCR protocols described above were conducted on a 2720 thermal cycler (Applied Biosystems, CA, USA). Reaction mixes included 2.5 units of MyTAQ™ DNA polymerase (Bioline GmbH, Luckenwalde, Germany), and 5× My*Taq*™ Reaction Buffer containing 5 mM dNTPs and 15 mM MgCl_2_. Laboratory PCR-confirmed positive and negative DNA samples for *G. duodenalis* or *Blastocystis* sp. were used as controls and included in each round of PCR. PCR amplicons were visualised on 2% D5 agarose gels (Conda, Madrid, Spain) stained with Pronasafe nucleic acid staining solution (Conda). Amplicons of the expected size were directly sequenced in both directions using the internal primer set described above. DNA sequencing was conducted by capillary electrophoresis using an Applied Biosystems*®* ABI PRISM 3130 automated DNA analyser at the Core Genomic Facility of the Spanish National Centre for Microbiology, Majadahonda, Spain*.* PCR and/or sequencing reactions were repeated on samples for which genotyping was unsuccessful in the first instance.

### Data analyses

The chi-square test was used to compare protozoan and helminth infection rates in the investigated communities according to gender, age group, and place of residence. The Kruskal-Wallis test and the Wilcoxon rank-sum test were used to compare qPCR cycle threshold (C_q_) values in stool samples with light, moderate, or heavy burdens of *G. duodenalis* cysts. A probability (*P*) value < 0.05 was considered evidence of statistical significance. Parameters showing zero values were omitted from the analyses due to insufficient statistical power. Prevalence risk ratios (PRR) and their 95% confidence intervals (CI) were calculated to assess the association between potential risk factors considered in the individual data collection spreadsheets and infections/carriage by intestinal parasites. Data were analysed with the free software OpenEpi version 3.01 [41].

### Sequence and phylogenetic analyses

Raw sequencing data in both forward and reverse directions were visually inspected using the Chromas Lite version 2.1 sequence analysis program (http://chromaslite.software.informer.com/2.1/). The BLAST tool (http://blast.ncbi.nlm.nih.gov/Blast.cgi) was used to search for identity among sequences deposited in the National Center for Biotechnology Information (NCBI) public repository database. Multiple sequence alignment analyses with appropriate reference sequences were conducted using MEGA 6 to identify *G. duodenalis* assemblages and sub-assemblages [42]. The newly-generated *Blastocystis* sequences were submitted to the *Blastocystis 18S* database (http://pubmlst.org/blastocystis/) for sub-type calling and allele identification.

The evolutionary relationships among the identified *Giardia*-positive samples were inferred by a phylogenetic analysis using the Neighbor-Joining method in MEGA 6. The evolutionary distances were computed using the Kimura 2-parameter method and modelled with a gamma distribution. The reliability of the phylogenetic analyses at each branch node was estimated by the bootstrap method using 1000 replications. Representative sequences of different *G. duodenalis* assemblages and sub-assemblages were retrieved from the NCBI database and included in the phylogenetic analysis for reference and comparative purposes. Representative sequences obtained in the present study were deposited in GenBank under the accession numbers MG807884-MG807901 (*G. duodenalis*) and MG807902-MG807921 (*Blastocystis* sp.).

## Results

Stool samples were collected from a total of 766 participants during the period of study. The estimated sample size (*n* = 1000) could not be reached because of the dengue fever epidemic that struck the region at the moment of sampling, impairing the recruitment of volunteers. The main demographic features of the surveyed population are summarised in Tables 1 and 2. Age groups were shown according to WHO World Standard Population Distribution [43]. The male/female ratio was 0.99. Overall, 81.5% (624/766) of the investigated individuals were of paediatric (0–14 years) age, whereas the adolescent and young adults (15–44 years-old) and the adults (≥ 45 years-old) groups accounted for 11.3% (87/766) and 7.2% (55/766) of the enrolled volunteers, respectively. Regarding their origin, three out of four participants (572/766) were recruited in urban and peri-urban environments, with the remaining 25% (194/766) living in insular or isolated rural settings.

**Table 1:** Distribution of the population recruited in the present study (*n* = 766) by gender, Paranaguá, Paraná, Brazil, 2015–2016

Community	*N*	Gender	
		Male *n* (%)	Female *n* (%)
Ilha do Mel	46	18 (39.1)	28 (60.9)
Ilha do Teixeira	58	30 (51.7)	28 (48.3)
Ilha do Amparo	90	45 (50.0)	45 (50.0)
Jardim Esperança	221	114 (51.6)	107 (48.4)
Ilha dos Valadares	286	144 (50.3)	142 (49.7)
Ponta do Caju	65	31 (47.7)	34 (52.3)
Total	766	382 (49.9)	384 (50.1)

*Abbreviation*: *N* number of individuals

**Table 2:** Distribution of the population recruited in the present study (*n* = 766) by age group, Paranaguá, Paraná, Brazil, 2015–2016

Community	*N*	Age group^a^, *n* (%)												
		0–4	5–9	10–14	15–19	20–24	25–29	30–34	35–39	40–44	45–49	50–54	55–59	> 59
Ilha do Mel	46	7 (15.2)	4 (8.7)	2 (4.3)	4 (8.7)	0 (0.0)	1 (2.2)	7 (15.2)	5 (10.9)	5 (10.9)	3 (6.5)	3 (6.5)	1 (2.2)	4 (8.7)
Ilha do Teixeira	58	6 (10.3)	3 (5.2)	4 (6.9)	4 (6.9)	0 (0.0)	4 (6.9)	8 (13.8)	3 (5.2)	4 (6.9)	2 (3.4)	2 (3.4)	6 (10.3)	12 (20.7)
Ilha do Amparo	90	11 (12.2)	13 (14.4)	18 (20.0)	4 (4.4)	6 (6.7)	1 (1.1)	7 (7.8)	7 (7.8)	6 (6.7)	2 (2.2)	5 (5.6)	4 (4.4)	6 (6.7)
Jardim Esperança	221	18 (8.1)	162 (73.3)	38 (17.2)	1 (0.5)	0 (0.0)	0 (0.0)	2 (0.9)	0 (0.0)	0 (0.0)	0 (0.0)	0 (0.0)	0 (0.0)	0 (0.0)
Ilha dos Valadares	286	23 (8.0)	187 (65.4)	63 (22.0)	1 (0.3)	1 (0.3)	1 (0.3)	0 (0.0)	2 (0.7)	3 (1.0)	0 (0.0)	1 (0.3)	0 (0.0)	4 (1.4)
Ponta do Caju	65	1 (1.5)	43 (66.2)	21 (32.3)	0 (0.0)	0 (0.0)	0 (0.0)	0 (0.0)	0 (0.0)	0 (0.0)	0 (0.0)	0 (0.0)	0 (0.0)	0 (0.0)
Total	766	66 (8.6)	412 (53.8)	146 (19.1)	14 (1.8)	7 (0.9)	7 (0.9)	24 (3.1)	17 (2.2)	18 (2.3)	7 (0.9)	11 (1.4)	11 (1.4)	26 (3.4)

*Abbreviation*: *N* number of individuals

^a^For practical purposes the age group > 59 years is an aggregate of the age groups 60–64, 65–69, 70–74, 75–79, 80–84, 85–89, 90–94, 95–99 and 100+ years

Table 3 shows relevant clinical characteristics and infection status of the individuals participating in the present study at the time of sampling. Regarding clinical signs typically associated with gastrointestinal disorders, abdominal pain and persistent diarrhoea were reported by 30.7% (235/766) and 13.3% (102/766) of the participants, respectively. Interestingly, the occurrence of both symptoms was evenly distributed among the investigated population irrespective of the gender or the age group considered. However, the frequency of diarrhoeal and/or abdominal pain episodes varied significantly among communities, with rates ranging between 6.9–21.1% and 24.8–49.2%, respectively. As expected in a community survey, most (69.3%; 531/766) of the stool samples obtained were formed. Watery faecal material consistent with acute diarrhoea was obtained in less than 5% of the cases. No significant associations between stool consistency and gender, age group or community of origin of the participants could be demonstrated.

**Table 3:** Main clinical parameters at the time of sampling of the individuals recruited in the present study (*n* = 766) according to their community of origin, gender and age group, Paranaguá, Paraná, Brazil, 2015–2016

Variable	*N*	Main symptomps, *n* (%)		Stool consistency, *n* (%)		
		Diarrhoea	Abdominal pain	Watery	Loose	Formed
Community						
Ilha do Mel^a^	46	8 (17.4)	13 (28.3)^4**^	2 (4.3)	16 (34.8)	28 (60.9)
Ilha do Teixeira^a^	58	4 (6.9)^1*^	16 (27.6)^5**^	2 (3.4)	11 (19.0)	45 (77.6)
Ilha do Amparo^a^	90	19 (21.1)^1*, 2*^	37 (41.1)^8**^	4 (4.4)	27 (30.0)	59 (65.6)
Jardim Esperança^b^	221	37 (16.7)^3*^	66 (29.9)^7**^	9 (4.1)	66 (29.9)	146 (66.0)
Ilha dos Valadares^b^	286	26 (9.1)^2*, 3*^	71 (24.8)^6**, 7**, 8**^	15 (5.2)	66 (23.1)	205 (71.7)
Ponta do Caju^b^	65	8 (12.3)	32 (49.2)^4**, 5**, 6**^	4 (6.1)	13 (20.0)	48 (73.9)
Gender						
Male	382	55 (14.4)	122 (31.9)	19 (5.0)	105 (27.5)	258 (67.5)
Female	384	47 (12.2)	113 (29.4)	17 (4.4)	94 (24.5)	273 (71.1)
Age group (years)						
0–4	66	6 (9.1)	15 (22.7)	5 (7.6)	15 (22.7)	46 (69.7)
5–9	412	58 (14.1)	127 (30.8)	24 (5.8)	108 (26.2)	280 (68.0)
10–14	146	21 (14.4)	52 (35.6)	5 (3.4)	44 (30.2)	97 (66.4)
15–34	52	6 (11.5)	16 (30.8)	1 (1.9)	12 (23.1)	39 (75.0)
> 34	90	11 (12.2)	25 (27.8)	1 (1.1)	20 (22.2)	69 (76.7)
Total	766	102 (13.3)	235 (30.7)	36 (4.7)	199 (26.0)	531 (69.3)

*Abbreviation*: *N* number of individuals

*P < 0.05, **P < 0.01: Statistically significant relationships between two given data for a specific variable were identified with superscript (1 to 8) numbers.

^a^Insular or poorly accessible settings

^b^Urban or peri-urban settings

### Prevalence estimates of intestinal parasites by microscopy

Coprological examination revealed the presence of protozoan and helminth species in 42.7% (327/766) and 10.1% (77/766) of the stool samples analysed, respectively. Overall, 46.1% (353/766) of the participants harboured at least one intestinal parasite/commensal species (Table 4). Among protists, these included pathogenic *Giardia duodenalis*, species of uncertain pathogenicity such as *Blastocystis* sp., and members of the *Entamoeba* complex (pathogenic *E. histolytica* and non-pathogenic, but morphologically indistinguishable, *E. dispar* and *E. moshkovskii*), and several commensal species including *Endolimax nana*, *Iodamoeba bütschlii*, *Entamoeba coli*, *Entamoeba hartmanni* (cyst averaged 7.1 ± 0.2 μm in diameter), *Chilomastix mesnilii*, and *Retortamonas intestinalis*. *Blastocystis* sp. was significantly (28.2%; *P* < 0.01) more frequently found than other protist species in the studied population, followed by *E. nana* (14.9%) and *G. duodenalis* (11.0%). All remaining microorganisms were observed at rates lower than 5%, whereas *Cryptosporidium* spp. was not detected. Helminth infections were comparatively less frequent (10.1%; 77/766), with *A. lumbricoides* (5.0%) and *T. trichiura* (4.6%) being the species more commonly identified. *Strongyloides stercoralis* and *Enterobius vermicularis* were only sporadically (≤ 1% of the stool samples examined) reported.

**Table 4:** Prevalence of enteric parasites in the recruited population (*n* = 766) in the present study, Paranaguá, Paraná, Brazil, 2015–2016

Parasite species	*N*	Prevalence (95% CI) (%)
Protozoans	327	42.7 (39.2–46.2)
*Entamoeba coli*	32	4.2 (2.9–5.8)
*Entamoeba hartmanni*	31	4.0 (2.8–5.6)
*Entamoeba* complex^a^	14	1.8 (1.0–3.0)
*Endolimax nana*	114	14.9 (12.5–17.5)
*Iodamoeba bütschlii*	5	0.7 (0.2–1.4)
*Giardia duodenalis*	84	11.0 (8.9–13.3)
*Chilomastix mesnilii*	3	0.4 (0.1–1.1)
*Retortamonas intestinalis*	2	0.3 (0.0–0.9)
*Blastocystis* sp.	216	28.2 (25.1–31.5)
Helminths	77	10.1 (8.1–12.3)
*Enterobius vermicularis*^b^	3	0.4 (0.1–1.1)
*Trichuris trichiura*	35	4.6 (3.3–6.2)
*Ascaris lumbricoides*	38	5.0 (3.6–6.7)
Family Ancylostomatidae	8	1.0 (0.5–2.0)
*Strongyloides stercoralis*	2	0.3 (0.0–0.9)
Total	353	46.1 (42.6–49.6)

*Abbreviations*: *N* number of infected individuals, *CI* confidence interval

^a^*Entamoeba* complex: *E. histolytica*/*E. dispar*/*E. moshkovskii*

^b^Diagnosis based exclusively on stool sample examination. No “Scotch tape” test was conducted

A significant difference was detected in the frequency that protozoan enteric parasites were detected in individuals from insular or isolated settings (50.5%, 98/194) compared to subjects from urban and peri-urban areas (40.0%, 229/572) (*P* = 0.011). In contrast, people living in urban and peri-urban areas were at higher risk (*P* = 0.038) of harbouring helminth infections (11.4%, 65/572) compared to those living in non-urban areas (6.2%, 12/194). Table 5 shows the infection rates by individual intestinal protozoan species according to the origin, gender, and age group of the recruited participants. Statistically significant differences were noticed among some of the variables considered. Infections with *Blastocystis* sp. were more prevalent in Ilha do Amparo (41.1%) and Ponta do Caju (35.4%), whereas *E. nana* was found at higher rates in Ilha do Teixeira (31.0%), and Ilha do Amparo (27.8%). Interestingly, *I. bütschlii* (2.3%) and *R. intestinalis* (1–2%) carriage was only observed in urban communities from Jardim Esperança and isolated human populations, respectively. Regarding gender, males exhibited higher infection rates by *G. duodenalis* than females (13.4 *vs* 8.6%; *P* = 0.036). The opposite trend was observed for *E. coli* (3.5 *vs* 5.7%; *P* = 0.031). Giardiasis showed a clear decreasing trend with age, with children nine-years-old and younger being more susceptible to the infection. In contrast, the prevalence of *E. nana* remained virtually constant in individuals in the 10–34 age group and older (21.2–22.2%), rates that almost doubled those find in children aged 0–4 (12.1%) and 5–9 (10.4%) years.

**Table 5:** Prevalence and 95% confidence intervals (in parentheses) of the enteric protozoan species identified in stool samples from the investigated population (*n* = 766) by conventional microscopy, Paranaguá, Paraná, Brazil, 2015–2016

Variable	N	Pathogenic	Uncertain pathogenicity		Commensals					
		*Giardia duodenalis*	*Blastocystis* sp.	*Entamoeba* complex	*Endolimax nana*	*Iodamoeba bütschlii*	*Entamoeba coli*	*Entamoeba hartmanni*	*Chilomastix mesnilii*	*Retortamonas intestinalis*
Community										
Ilha do Mel^1^	46	6.5 (1.7–16.7)	28.3 (16.7–42.5)	6.5 (1.7–16.7)	15.2 (6.9–27.8)	0 (0.0–6.3)	6.5 (1.7–16.7)	6.5 (1.7–16.7)	0 (0.0–6.3)	2.2 (0.1–10.3)
Ilha do Teixeira^1^	58	3.4 (0.6–10.9)	22.4^a*^ (13.1–34.5)	0 (0.0–5.0)	31.0^d**, e**, f**^ (20.2–43.8)	0 (0.0–5.0)	3.4 (0.6–10.9)	0 (0.0–5.0)	0 (0.0–5.0)	0 (0.0–5.0)
Ilha do Amparo^1^	90	13.3 (7.4–21.6)	41.1^a*, b**^ (31.3–51.5)	0 (0.0–3.3)	27.8^g**, h**, i**^ (19.3–37.7)	0 (0.0–3.3)	3.3 (0.9–8.8)	4.4 (1.4–10.4)	1.1 (0.1–5.4)	1.1 (0.1–5.4)
Jardim Esperança^2^	221	14.5 (10.3–19.6)	32.6 (26.6–39)	2.7 (1.1–5.6)	14.5^d**, g**^ (10.3–19.6)	2.3 (0.8–4.9)	2.7 (1.1–5.6)	5.0 (2.6–8.5)	0.9 (0.0–2.3)	0 (0.0–1.3)
Ilha dos Valadares^2^	286	10.1 (7–14.1)	20.3^b**, c**^ (15.9–25.2)	1.0 (0.3–2.8)	8.7^e**, h**^ (5.9–12.5)	0 (0.0–1.0)	5.6 (3.3–8.7)	3.8 (2.0–6.6)	0 (0.0–1.0)	0 (0.0–1.0)
Ponta do Caju^2^	65	9.2 (3.8–18.2)	35.4^c**^ (24.5–47.5)	3.1 (0.5–9.8)	10.8^f**, i**^ (4.8–20.1)	0 (0.0–4.5)	3.1 (0.5–9.8)	3.1 (0.5–9.8)	0 (0.0–4.5)	0 (0.0–4.5)
Gender										
Male	382	13.4^j*^ (10.2–17)	28.0 (23.7–32.7)	1.8 (0.8–3.6)	14.9 (11.6–18.8)	0.8 (0.2–2.1)	3.5^k*^ (1.8–6.2)	3.9 (2.3–6.3)	0.5 (0.1–1.7)	0.3 (0–1.3)
Female	384	8.6^j*^ (6.1–11.7)	28.4 (24.0–33.1)	1.8 (0.8–3.6)	14.8 (11.6–18.7)	0.5 (0.1–1.7)	5.7^k*^ (3.7–8.4)	4.2 (2.5–6.5)	0.3 (0.0–1.3)	0.3 (0.0–1.3)
Age group (years)										
0–4	66	13.6^l*^ (6.9–23.6)	25.8 (16.3–37.3)	0 (0.0–4.4)	12.1 (5.8–21.7)	0 (0.0–4.4)	4.5 (1.2–11.9)	1.5 (0.1–7.2)	0 (0.0–4.4)	0 (0.0–4.4)
5–9	412	13.3^m**^ (10.3–16.9)	26.9 (22.8–31.4)	1.5 (0.6–3.0)	10.4^n**, o*, p**^ (7.8–13.7)	0.7 (0.2–2.0)	2.9 (1.6–4.9)	3.4 (2.0–5.5)	0.2 (0.0–1.2)	0 (0.0–0.7)
10–14	146	9.6 (5.6–15.2)	31.5 (24.4–39.4)	2.7 (0.9–6.5)	21.9^p**^ (15.7–29.2)	1.4 (0.2–4.5)	8.2 (4.5–13.6)	6.8 (3.5–11.9)	1.4 (0.2–4.5)	0.7 (0.0–3.3)
15–34	52	5.8 (1.5–14.9)	30.8 (19.4–44.2)	1.9 (0.1–9.1)	21.2^o*^ (11.7–33.8)	0 (0.0–7.0)	5.8 (1.5–14.9)	3.8 (0.7–12.1)	0 (0.0–5.6)	0 (0.0–5.6)
> 34	90	3.3^l*, m**^ (0.9–8.8)	28.9 (20.2–38.9)	3.3 (0.9–8.8)	22.2^n**^ (14.5–31.7)	0 (0.0–3.5)	2.2 (0.4–7.1)	4.4 (1.4–10.4)	0 (0.0–3.3)	1.1 (0.1–5.4)
Total	766	11.0 (8.9–13.3)	28.2 (25.1–31.5)	1.8 (1.0–3.0)	14.9 (12.5–17.5)	0.7 (0.2–1.4)	4.2 (2.9–5.8)	4.0 (2.8–5.6)	0.4 (0.1–1.1)	0.3 (0.0–0.9)

*Abbreviation*: *N* number of samples

*P < 0.05, **P < 0.01: Statistically significant relationships between two given data for a specific variable were identified with superscript (a to p) letters

^1^Insular or poorly accessible settings

^2^Urban or peri-urban settings

Table 6 shows the prevalence of intestinal helminth infections according to the origin, gender, and age group of the surveyed population. As in the case of enteric protozoan species, a number of statistically significant differences were demonstrated. Of note, *A. lumbricoides* was only detected in subjects living in urban and peri-urban settings, but this soil-transmitted nematode was absent from insular communities. A similar situation was observed for *T. trichiura*, with the exception that trichuriasis was also reported in a significant proportion (7.8%) of individuals living in Ilha do Amparo. In contrast, members of the family Ancylostomatidae were primarily found in isolated communities rather than in urban or peri-urban areas. Gender was not a predictor of infection by any of the helminth species reported here. Finally, age-related infection patterns were observed for some of STHs detected. This was the case for *A. lumbricoides*, found at increasing infection rates in individuals of paediatric age but not identified in subjects older than 14 years. An age-cumulative increase on the prevalence of ancylostomids was also noted through all age groups considered except for that of > 34 years-old. Because *S. stercoralis* and *E. vermicularis* were only sporadically identified, no clear geographical or age-related distribution patterns could be elucidated.

**Table 6:** Prevalence and 95% confidence intervals (in parentheses) of the enteric helminth species identified in stool samples from the investigated population (*n* = 766) by conventional microscopy, Paranaguá, Paraná, Brazil, 2015–2016

Variable	*N*	*Ascaris lumbricoides*	*Trichuris trichiura*	Ancylostomatidae	*Strongyloides* spp.	*Enterobius vermicularis*
Community						
Ilha do Mel^a^	46	0 (0.0–6.3)	0 (0.0–6.3)	2.2 (0.1–10.3)	0 (0.0–6.3)	0 (0.0–6.3)
Ilha do Teixeira^a^	58	0 (0.0–5.0)	0 (0.0–5.0)	0 (0.0–5.0)	1.7 (0.1–8.2)	0 (0.0–5.0)
Ilha do Amparo^a^	90	0 (0.0–3.3)	7.8^2**^ (3.5–14.8)	6.7^4**^ (2.7–13.4)	1.1 (0.1–5.4)	0 (0.0–3.3)
Jardim Esperança^b^	221	6.3 (3.7–10.2)	0.9^1**, 2**, 3**^ (0.2–3.0)	0.5^4**^ (0.0–2.2)	0 (0.0–1.3)	0.5 (0.0–2.2)
Ilha dos Valadares^b^	286	7.7 (5–11.2)	7.0^3**^ (4.4–10.4)	0 (0.0–1.0)	0 (0.0–1.0)	0.7 (0.1–2.3)
Ponta do Caju^b^	65	3.1 (0.5–9.8)	9.2^1**^ (3.8–18.2)	0 (0.0–4.5)	0 (0.0–4.5)	0 (0.0–4.5)
Gender						
Male	382	6.0 (3.9–8.8)	6.0 (3.9–8.8)	1.6 (0.6–3.2)	0.5 (0.1–1.7)	0.7 (0.1–2.3)
Female	384	3.9 (2.3–6.2)	3.1 (1.7–5.3)	0.5 (0.1–1.7)	0 (0.0–0.8)	0.4 (0.0–1.7)
Age group (years)						
0–4	66	3.0 (0.5–9.7)	1.5 (0.1–7.2)	0 (0.0–4.4)	0 (0.0–4.4)	0 (0.0–4.4)
5–9	412	5.8 (3.9–8.4)	5.6 (3.7–8.1)	0.2 (0.0–1.2)^5**, 6*^	0 (0.0–0.7)	0.7 (0.2–2.0)
10–14	146	8.2 (4.5–13.6)	6.2 (3.0–11.0)	1.4 (0.2–4.5)	0 (0.0–2.0)	0 (0.0–2.0)
15–34	52	0 (0.0–6.0)	0 (0.0–6.0)	5.8^5**^ (1.5–14.9)	0 (0.0–6.0)	0 (0.0–5.6)
> 34	90	0 (0.0–3.2)	2.2 (0.4–7.1)	2.2^6*^ (0.4–7.1)	2.2 (0.4–7.1)	0 (0.0–3.3)
Total	766	5.0 (3.6–6.7)	4.6 (3.3–6.2)	1.0 (0.5–2)	0.3 (0.0–0.9)	0.4 (0.1–1.1)

*Abbreviation*: *N* number of samples

*P < 0.05, **P < 0.01: Statistically significant relationships between two given data for a specific variable were identified with superscript (1 to 6) numbers

^a^Insular or poorly accessible settings

^b^Urban or peri-urban settings

### Parasite infection intensities

**Fig. 2:**
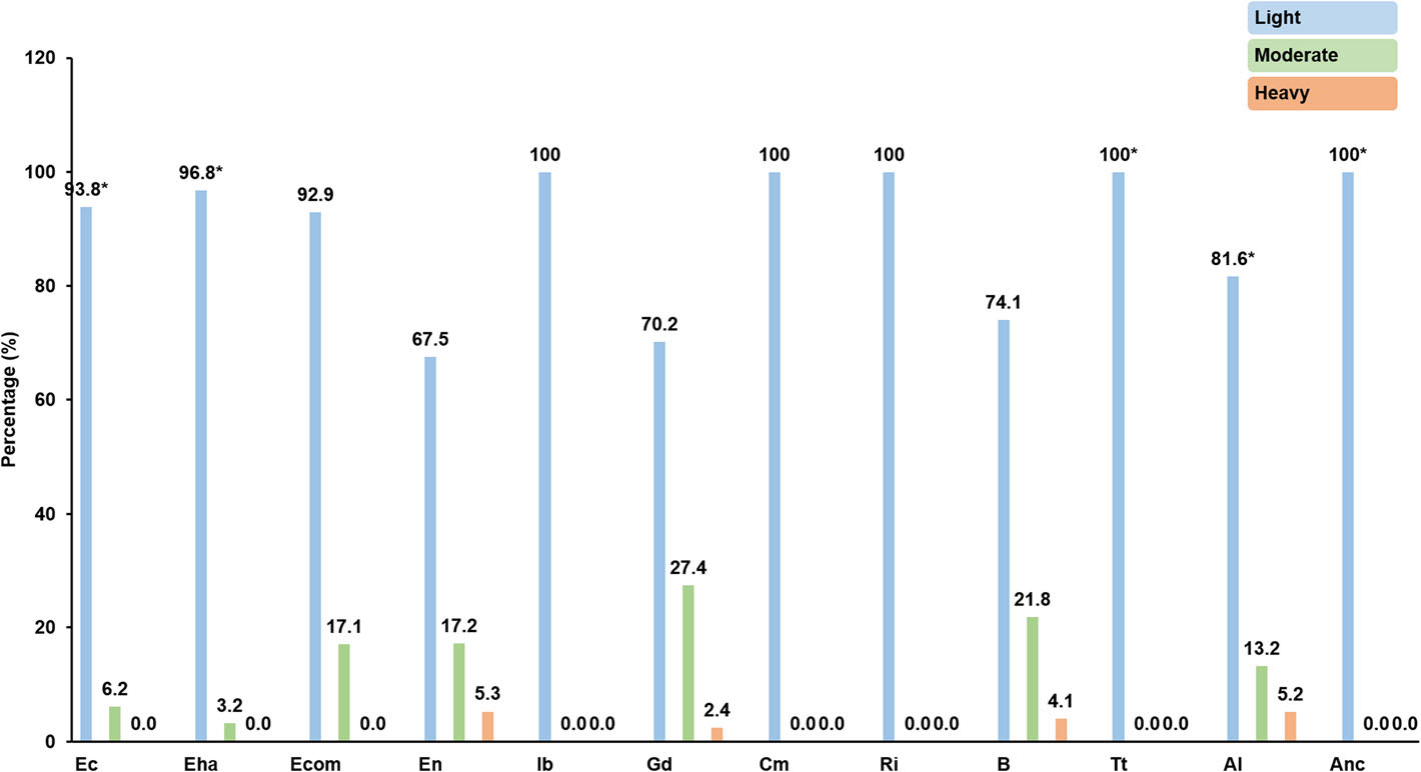
Distribution of individual protozoa and helminth species according to their estimated infection intensities. *Abbreviations*: Ec, *Entamoeba coli*; Eha, *Entamoeba hartmanni*; Ecom, *Entamoeba* complex (*Entamoeba histolytica*/*Entamoeba dispar*/*Entamoeba moshkovskii*); En, *Endolimax nana*; Ib, *Iodamoeba bütschlii*; Gd, *Giardia duodenalis*; Cm, *Chilomastix mesnili*; Ri, *Retortamonas intestinalis*; B, *Blastocystis* sp.; Tt, *Trichuris trichiura*; Al, *Ascaris lumbricoides*; Anc, family Ancylostomatidae. Statistical significance (*P* < 0.01) is indicated by asterisks

A total of 501 infections with individual species were detected in the 327 stool samples that tested positive for any given intestinal parasite during microscopic examination. Overall, three out of four (379/501) infections were considered of low intensity, whereas moderate and high infections were observed in 21.0% (105/501) and 3.4% (17/501) of the cases, respectively. The distribution of individual protozoa and helminth species according to their estimated infection intensities is shown in Fig. 2.

The relationship between infection intensity and occurrence of compatible symptomatology (prolonged diarrhoeal episodes and abdominal pain) at the time of sampling was investigated for *G. duodenalis* and *Blastocystis* sp., two of the most prevalent protozoan species in the surveyed population. Interestingly, individuals with low (44.1 *vs* 26.2%, *P* = 0.001) or moderate (22.6 *vs* 4.7%, *P* < 0.001) burdens of *G. duodenalis* had a higher likelihood of reporting gastrointestinal symptoms, whereas the cases with high burdens of the parasite were equally distributed between symptomatic and asymptomatic subjects (1.2% each). Because of the unclear pathogenic role of *Blastocystis*, only mono-infected individuals were considered in the analysis. In that particular sub-population, *Blastocystis* sp. carriage at low intensity was significantly higher (*P* < 0.001) in asymptomatic than symptomatic (51.7 *vs* 28.1%) participants. Moderate burdens of the parasite were more frequently observed in symptomatic (10.2%) than in asymptomatic (5.6%) cases, although without reaching statistical significance. Although we cannot rule out the possibility that some of the clinical symptoms observed in these individuals were due to viral or bacterial agents (pathogens not investigated in the present survey), the fact that most of the subjects complaining of gastrointestinal illness presented with persistent diarrhoea seems to suggest a parasitic rather than a bacterial/viral infection. As in the case of *G. duodenalis*, high burdens of *Blastocystis* sp. were equally observed in both symptomatic and asymptomatic subjects.

### Single and multiple intestinal protozoan and helminthic infections

The frequencies of single and multiple infections involving enteric protozoan and helminth parasites in the studied population are shown in Additional file 2: Table S2. Monoparasitism (55.0%, 194/353) was more prevalent than double (28.9%, 102/353), triple (11.9%, 42/353), quadruple (3.4%, 12/353) and quintuple (0.8%, 3/353) infections, respectively. Based on their frequency of occurrence, *Blastocystis* sp. (25.2%, 89/353), *G. duodenalis* (8.8%, 31/353) and *E. nana* (9.9%, 35/353) were the parasitic species more often found in single infections. Similarly, *Blastocystis* sp. in combination with *E. nana* (7.6%, 27/353), *G. duodenalis* (5.4%, 19/353) and *E. coli* (2.3%, 8/353) were the combinations more commonly identified in double infections. The most frequent triple infections observed included *Blastocystis* sp. and *E. nana* in combination with *E. hartmanni* (2.0%, 7/353), *G. duodenalis* (1.7%, 6/353) and *E. coli* (1.7%, 6/353). A total of 10 quadruple and three quintuple parasitic combinations were also found (Additional file 2: Table S2).

### Molecular identification and characterisation of *G. duodenalis* isolates

**Fig. 3:**
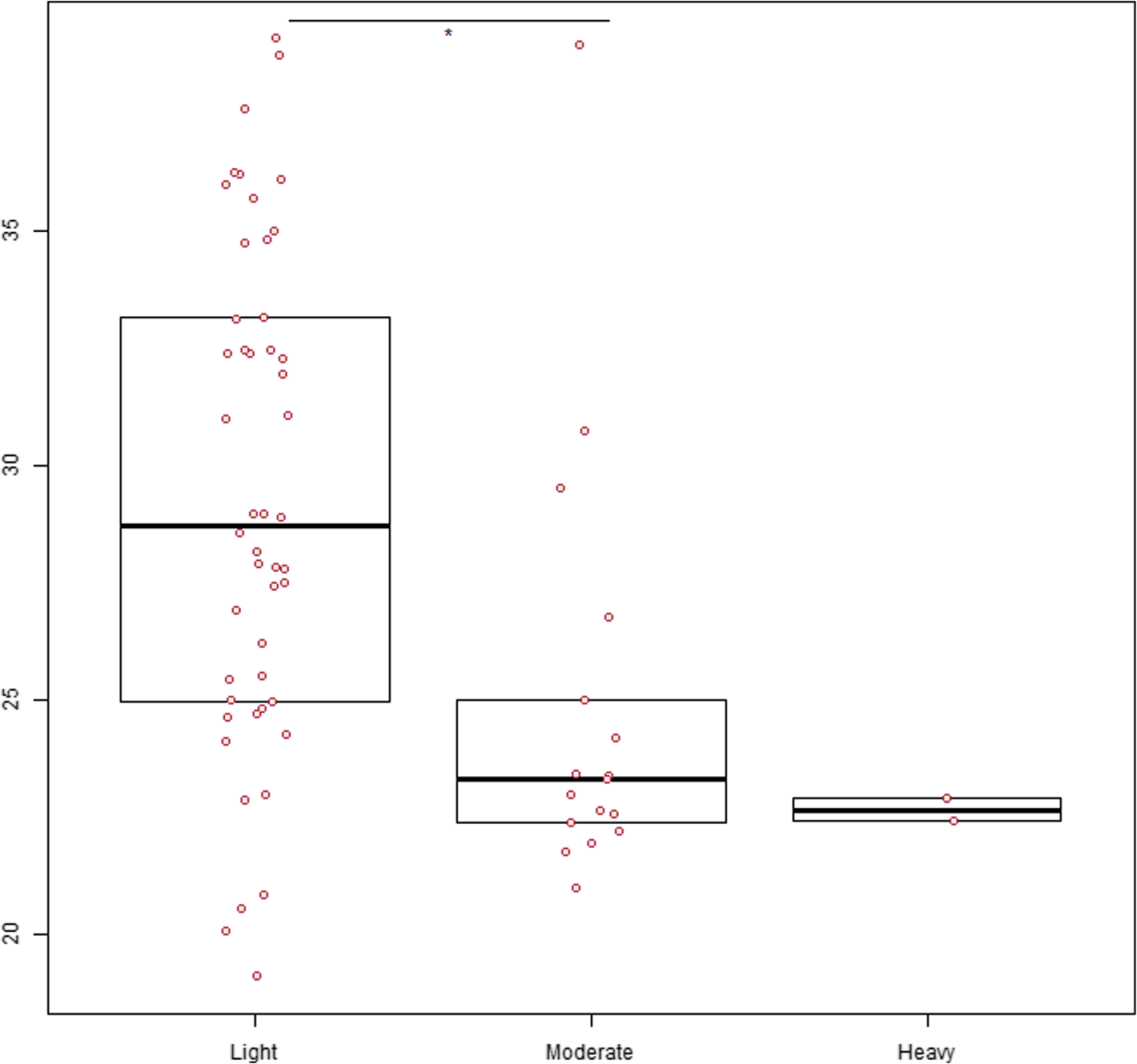
Dot-and-box plots showing the distribution of the cycle threshold values obtained by qPCR in *G. duodenalis*-positive samples according to their infection intensity as estimated by microscopy. The bottom and top lines of the boxes indicate the first and third quartiles, and the thicker line inside the box represents the second quartile (the median). Statistical significance (*P* < 0.01) is indicated by asterisks

Of the 84 stool samples with a positive result for *G. duodenalis* by microscopy, a total of 72 were available for genomic DNA extraction, purification, and subsequent downstream molecular analyses. Quantitative real-time PCR confirmed the presence of the parasite in 93.1% (67/72) of the cases. Obtained C_q_ values ranged from 19.1–39.1 (median: 28.0; SD: 5.4). As expected, obtained C_q_ values (indicative of the starting amount of target DNA) were inversely proportional to the number of *G. duodenalis* cysts (infection intensity) recorded during the microscopic examination (Fig. 3). Indeed, the Kruskal-Wallis test showed that there was a statistically significant difference (*χ*^2^ = 13.39, *df* = 2, *P* < 0.01) among the burden categories (light, moderate and heavy) considered. When the Wilcoxon rank-sum test was used, these differences were demonstrated between the light and moderate burden categories (*W* = 628, *P* < 0.001).

Out of the 72 *G. duodenalis* isolates obtained, 50.0% (36/72) and 19.4% (14/72) were successfully amplified at *gdh* and *bg* loci, respectively. Thirty-eight isolates were genotyped and/or sub-genotyped by any of the two markers, whereas multi-locus sequence typing data were available for 16.7% (12/72) of the isolates characterised. DNA amplification success rates for both *gdh*-PCR and *bg*-PCR were found to be highly dependent on qPCR C_q_ values. Only a single *gdh* and two *bg* PCR amplicons were obtained from *G. duodenalis* isolates with qPCR C_q_ values > 30, which represented 37.5% (27/72) of the total.

Table 7 shows the diversity, frequency and main features of the *G. duodenalis* sequences generated at *gdh* and *bg* markers in the present study. Sequence analyses revealed that assemblages A and B were equally represented (50.0%, 19/38 each) in the human population under study. No indirect evidence of zoonotic transmission events involving canine (C, D), feline (F), or ruminant (E) assemblages of *G. duodenalis*, nor inter-assemblage (A + B) mixed infections were detected. Further sub-genotyping analyses (see below) revealed that, out of the 19 assemblage A sequences, most of them (94.7%, 18/19) corresponded to the sub-assemblage AII. Ten of these AII isolates were confirmed both at *gdh* and *bg* loci, and eight at the former locus only. Ambiguous AII/AIII results were obtained for a single (5.3%; 1/19) isolate at both the *gdh*/*bg* loci. Regarding the 19 isolates of *G. duodenalis* assemblage B obtained, 10.5% (2/19) and 52.7% (10/19) were assigned to the sub-assemblages BIII and BIV, respectively, at *gdh* only. Ambiguous BIII/BIV results were generated in 26.3% (5/19) of the cases (four at *gdh* and one at *gdh* and *bg* loci), whereas the remaining 10.5% (2/19) of the isolates were characterised only at the assemblage level at *bg*, but not *gdh*, locus. Sub-assemblage AII was significantly more prevalent both in males (52.6%, *P* = 0.001) and females (47.1%, *P* < 0.001) than other sub-assemblages of the parasite. Sub-assemblage BIV was predominantly detected in children aged 0–4 years-old (75.0%, *P* = 0.031), whereas AII was the predominant *G. duodenalis* sub-assemblage in the age groups 5–9 years (45.8%, *P* = 0.002) and 10–14 years (75.0%, *P* < 0.001). Interestingly, individuals harbouring the sub-assemblage AII of the parasite were more likely to report recent episodes of gastrointestinal disorders, mainly persistent diarrhoea and/or abdominal pain.

**Table 7:** Diversity, frequency, and molecular features of *Giardia duodenalis* sequences at *gdh* and *bg* loci obtained in the children population under study, Paranaguá, Paraná, Brazil, 2015–2016

Locus	Assemblage	Sub-assemblage	No. of isolates	Reference sequence	Stretch	Single nucleotide polymorphisms	GenBank ID
*gdh*	A	AII	17	L40510	78–481	None	MG807884
		AII	1	L40510	78–481	C310Y	MG807885
	B	BIII	1	AF069059	40–460	C171Y, T456Y	MG807886
			1	AF069059	43–460	C309T, T456Y	MG807887
		BIV	2	L40508	76–447	None	MG807888
			1	L40508	76–496	G84R, G156R, T183Y, G294R, C345Y, T387Y, C432Y, G453R	MG807889
			1	L40508	76–491	G93A, C123T, T135C, C273T	MG807890
			4	L40508	80–476	G180A	MG807891
			1	L40508	76–447	T183C, T387C, C432T	MG807892
		BIII/BIV	1	L40508	80–485	T135Y, T183Y, G186R, C255Y, C273Y, C345Y, T366Y, C372Y, T387C, C396Y, C423Y, A438R	–
			1	L40508	80–485	T183Y, C255Y, C273Y, T366Y, T387C, C396T, C423Y, A438R	–
			1	L40508	80–480	T135Y, T183Y, C255Y, C273Y, G334R, C345Y, T366Y, T387C, C396Y, A438R	–
			1	L40508	78–482	C123Y, T135Y, T183C, G186R, C255Y, C273Y, C345Y, T366Y, C372Y, T387C, A438R, T462Y	MG807893
			1	L40508	80–485	T135Y, C192Y, C255Y, C258Y, C273Y, C345Y, T366Y, C372Y, T387Y, A438R, T462Y	–
*bg*	A	AII	6	AY072723	102–590	None	MG807893
			1	AY072723	102–589	224delA	MG807895
			1	AY072723	102–603	G277R	MG807896
			1	AY072723	127–586	T469Y	MG807897
		AIII	1	AY072724	102–590	G322A^a^, A457G^b^, A463G^c^	MG807898
	B		1	AY072727	105–590	None	MG807899
			1	AY072727	103–590	T209Y	MG807900
			1	AY072727	102–590	C309T, T519Y, C564Y, C567Y	MG807901

Novel genotypes are underlined. Point mutations inducing amino acid substitutions are indicated with superscript letters indicating the amino acid change

R: A/G; Y: C/T

^a^p. D108N

^b^p. R153G

^c^p. K155E

**Fig. 4:**
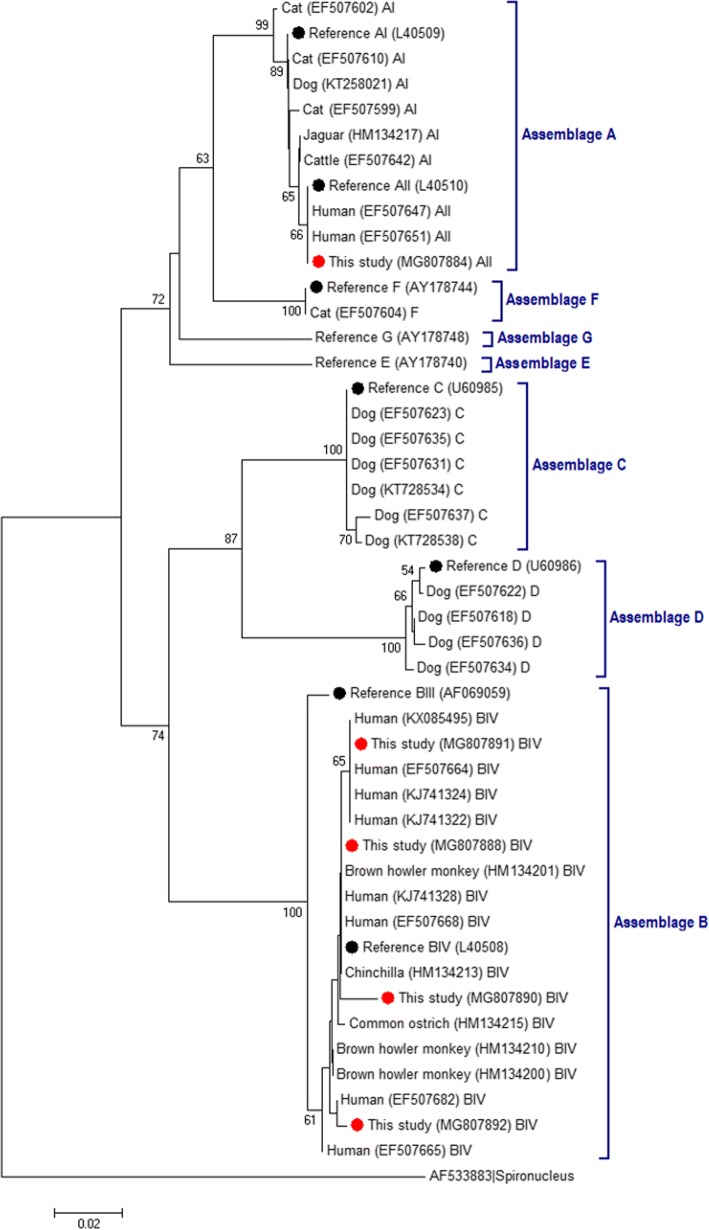
Phylogenetic tree depicting evolutionary relationships among *Giardia duodenalis* sequences at *gdh* from Brazilian human and animal isolates. The analysis was inferred using the Neighbor-Joining method of the nucleotide sequence covering a 388-bp region (positions 80–467 of GenBank: L40508) of the gene. Bootstrap values lower than 50% are not shown. Red circles represent sequences generated in the present study; black circles represent reference sequences downloaded from the GenBank database. *Spironucleus vortens* was used as the outgroup

At *gdh*, all *G. duodenalis* sub-assemblage AII isolates exhibited very high sequence homogeneity. Sequence alignment analysis revealed that 17 out of the 18 AII isolates showed 100% identity with a 404-bp fragment equivalent to positions 80–478 of the corresponding reference sequence (GenBank: L40510). The remaining AII isolate differed from L40510 by a single-nucleotide polymorphism (SNP) at position 310 (Table 7). In contrast, a much higher degree of genetic diversity was observed among the *G. duodenalis* sub-assemblage BIII and BIV isolates (particularly the latter) at this specific locus. For instance, the two BIII sequences generated in this survey differed by two SNPs, and both differed by three SNPs from the reference sequence AF069059 between positions 40/43–460. Nucleotide heterogeneity was even more apparent among BIV isolates, where a total of five distinct genotyping profiles were obtained (Table 7). Two of the BIV sequences were identical to the stretch comprising positions 76 to 447 of reference sequence L40508, while another four BIV sequences differed only by a single SNP (G to A) at position 180 of L40508. The remaining three isolates corresponded to distinct BIV genetic variants (including the novel genotype MG807890) varying from three to eight SNPs in a stretch comprising positions 76 to 447/496 of reference sequence L40508 (Table 7). Additionally, multiple alignments of discordant BIII/BIV sequences with reference sequences AF069059 (BIII) and L40508 (BIV) revealed the presence of 8–12 SNPs. Most sequence variations occurred at discrete positions and mostly involved heterozygous bases in the form of double peaks in the electropherograms, strongly suggesting the presence of BIII + BIV mixed infections (Table 7). Figure 4 shows the phylogenetic relationships among representative, unambiguous (homozygous) sequences at *gdh* generated in this survey and reference sequences from NCBI. Publicly available sequences of Brazilian isolates of human and animal (livestock, companion, wildlife) species were also included in the analysis for comparative purposes. As expected, sequences assigned to assemblages A and B grouped in distinct clusters. Reflecting their comparatively elevated rate of nucleotide substitutions per site, the novel (MG807890) and known (MG807892) BIV sequences exhibited more considerable branch lengths than previously reported Brazilian human isolates of *G. duodenalis* sub-assemblage BIV.

Confirming initial information generated at *gdh*, AII sequences obtained at *bg* also showed little molecular diversity at the nucleotide level. Six out of nine AII sequences were identical to the stretch comprising positions 102 to 590 of reference sequence AY072723 (Table 7). The remaining three isolates corresponded to two known and a novel (MG807895) genotypes, the latter involving an A deletion at position 224 of AY072723. Of interest, a single isolate previously characterised as AII at *gdh* was confirmed as AIII at *bg*. A more in in-depth sequence analysis revealed that this isolate (MG807898) represented a novel AIII genotype with three SNPs associated with amino acid substitutions in the protein chain. Finally, out of the three assemblages, B isolates amplified at *bg*, one showed 100% sequence identity with a stretch covering positions 105 to 590 of reference sequence AY072727. The remaining two sequences differed from AY072727 by one to four SNPs mostly associated to mixed bases (double peaks).

### Molecular identification and characterisation of *Blastocystis* sp. isolates

**Fig. 5:**
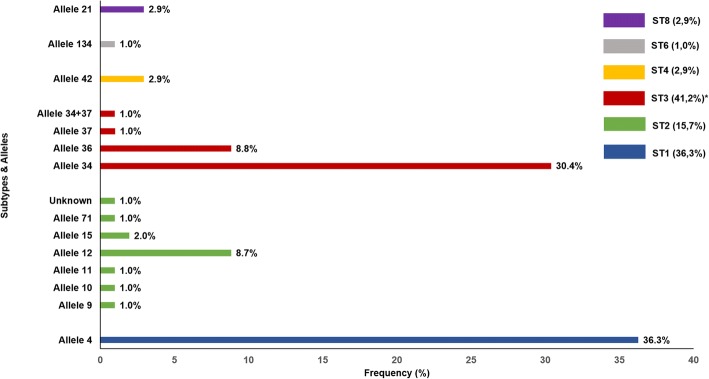
Diversity and frequency of *Blastocystis* subtypes and 18S alleles identified in the children population under study, Paranaguá, Paraná, Brazil, 2015–2016. Statistical significance (*P* < 0.01) is indicated by asterisks

Of the 216 samples that tested positive for *Blastocystis* sp. by microscopy, genomic DNA was only available for 147 samples due to faecal material preservation issues. Of them, 80.3% (118/147) were confirmed by PCR, and sequence analyses successfully subtyped 69.4% (102/147) at the *SSU* rRNA (barcode region) gene. A total of 14 samples were untypable due to poor sequence quality. BLAST searches of the two additional isolates, which differed by a single SNP, revealed significant similarity (92%) with *Blastocystis lapemi*, a *Blastocystis* species firstly described in sea snakes of the genus *Lapemis* [44]. Additional analyses are being conducted in our laboratory to generate the full-length *SSU* rDNA sequences of these isolates and accurately determine their actual taxonomic identity. Typable isolates were unambiguously assigned to ST1 (36.3%; 37/102), ST2 (15.7%; 16/102) and ST3 (41.2%; 42/102). Less prevalent subtypes included ST4 (2.9%; 3/102), ST6 (1.0%; 1/102) and ST8 (2.9%; 3/102) (Fig. 5). Neither mixed infection involving different STs of the parasite nor infections caused by animal-specific ST10-ST17 were identified. Males were significantly more prone to carry ST3 (45.3%; *P* < 0.001), whereas ST1 was more likely to be found in females (40.8%; *P* < 0.001). Additionally, ST3 carriage was predominantly detected in individuals belonging to age groups 0–4 (66.7%) and 5–9 (43.3%), and ST1 in subjects between 10–14 years of age (*P* < 0.001).

Allele calling using the *Blastocystis SSU* database revealed the presence of allele 4 within ST1, alleles 9–12, 15 and 71 within ST2, alleles 34, 36 and 37 within ST3, allele 42 within ST4, allele 134 within ST6, and allele 21 within ST8 (Fig. 4). According to their frequency of occurrence, ST1 allele 4 (36.3%), ST3 alleles 34 (29.4%) and 36 (8.8%), and ST2 allele 12 (8.8%) were more prevalent in the surveyed population. A single ST2 isolate could not be analysed at the allele level due to low-quality sequence data. Additionally, and based on multiple sequence alignment analysis and chromatogram inspection, a mixed infection involving alleles 34+37 was identified in a *Blastocystis* ST3 isolate.

## Discussion

Intestinal parasites are among the most frequent pathogens infecting humans, causing gastrointestinal and nutritional disorders in institutional (e.g. day-care centres) and community settings both in developing and developed countries [2, 6]. Infections caused by EPs are commonly linked to poverty and have a significant impact on the socio-economic development of endemic and hyper-endemic areas [3, 4], including Brazil [45–47].

In the present survey, 46.1% of the volunteer community participants were found infected/colonised by at least one intestinal parasite/commensal species. Although still considerable, this figure is significantly lower than the 99.3% rate reported in 1962 by Lima et al. [27] and the 78.8% rate found in 2002 by Klisiowicz [28] in similar surveys in this geographical area. This marked a declining trend in prevalence rates and is very likely due to the improvements in drinking water sources and sanitation facilities and the mass treatment campaigns conducted in the Paraná State in the last decades. Remarkably, we observed that protozoan EPs were significantly more prevalent in individuals living in insular/isolated areas than those living in urban and peri-urban settings, whereas the opposite trend was noticed for helminth EPs. These findings seem to suggest the existence of different contamination sources and transmission pathways among the surveyed communities. In this regard, it would be exciting to investigate further whether differences in the microbiological quality of water sources and raw vegetables for human consumption may explain, at least partially, the observed distribution of protozoan EPs, as water- and food-borne transmission of these pathogens has been suggested in previous studies conducted in the Paraná State [48–51].

A wide diversity of intestinal protozoan (*n* = 9) and helminth (*n* = 5, four of them STHs) species were detected, most of them (68–100%) at light infection/carriage intensities. Among protozoa, we report here the first detection of *R. intestinalis* and *E. hartmanni* in the region. Regarding protozoan EPs of public health relevance, *Blastocystis* sp. (28.2%) and *G. duodenalis* (11.0%) were the species more frequently found. Both have been previously identified at prevalences typically ranging between 1–33% and 7–51%, respectively, in other Brazilian regions including Minas Gerais [52, 53], Rio Grande do Sul [54], Santa Catarina [55] and São Paulo [56, 57]. *Blastocystis* carriage was homogeneously distributed among all age groups investigated. This was in contrast with previous findings reporting that the presence of *Blastocystis* was positively associated with age, with colonisation being more common in older children and adults [52, 58, 59]. Regarding the potential pathogenicity of *Blastocystis* sp., it has been argued that symptoms may be linked to colonisation intensity [60–62], although such a correlation has not been confirmed in other surveys [63]. In the Paraná State, *Blastocystis* colonisation was more frequently found in patients undergoing haemodialysis (45.1%) than in apparently healthy (control) individuals (25.7%), a difference not observed for other enteric protozoan species including *E. nana*, *Cryptosporidium* spp. and *E. coli* [64]. In our study, symptomatic *Blastocystis* carriage was more frequently observed in individuals with moderate colonisation intensities than in those with light colonisation intensities, although without reaching statistical significance. Discrepancies due to symptom subjectivity during reporting may account for the differences observed in these investigations. *Giardia duodenalis* infections followed distinctive gender- and age-related distribution patterns, being significantly more frequent in males than in females and children younger than ten years of age than in older subjects. The observed predominance of giardiasis in male subjects may be linked to unidentified behavioural or occupational habits [65, 66]. Also, reporting of gastrointestinal symptoms was significantly higher in individuals with light to moderate *Giardia* infection burdens than in heavily infected individuals. Intriguingly, we could not detect the presence of *Cryptosporidium* spp. in the investigated population, even though this pathogen has been previously reported in water intended for irrigation purposes and in raw (but not treated) water for human consumption in the Paraná State [48, 50].

Helminth EPs were detected at much lower (≤ 5%) infection rates than those reported in earlier epidemiological surveys [27, 28], very likely due to the sanitary improvements (latrines, latrine maintenance, faecal sludge management) and mass-treatment and health education programmes carried out in the region commented above. Consequently, parasite species including *Schistosoma mansoni*, *Taenia* spp., and *Hymenolepis nana*, known to be present in the Paraná State years ago, were not identified in the present study. Because the small number of helminth-positive samples obtained in the surveyed population, the only factor associated to a higher risk of infection by helminths was gender, with male subjects being more likely to be infected than female individuals.

This survey also provides molecular data regarding the diversity and frequency of two of the most prevalent enteric protozoan species detected, namely *G. duodenalis* and *Blastocystis* sp. Regarding the former, more than half (52.8%) of the available positive samples were genotyped at *gdh* and/or *bg* loci. This success rate was considerably higher than those (21.1–36.1%) achieved by our research group using the same analytical tools and similar experimental design in studies conducted in Angola [67] and Ethiopia [68]. These discrepancies are associated to differences in basal parasite burden (and, therefore, starting parasite DNA concentrations) among the human populations investigated, as revealed by the obtained median qPCR C_q_ values (28.0 in the present study *vs* 31.5 and 33.2 in the Ethiopian and Angolan surveys, respectively). Overall, these data seem to suggest that *G. duodenalis* infections are common in the State of Paraná and occur at average intensities higher than those present in other endemic areas. Indeed, we have shown here that 36.0% of the infections by this protozoan species were classified as moderate.

*Giardia duodenalis* assemblages A and B were equally present in the population under study. Similar A/B proportions have been previously reported in individuals of paediatric age in the State of Santa Catarina [69], in Amerindian children in the State of Amazonas [70], in deprived communities in the State of São Paulo [57], and in patients attending a hospital setting in Rio de Janeiro [71]. Interestingly, the presence of assemblage A, but not assemblage B, has been described in a few epidemiological surveys targeting clinical patients in the State of São Paulo [72], and paediatric populations [73] and people from disadvantaged communities seeking medical care [74] in Rio de Janeiro. In contrast, assemblage B was more prevalently found in asymptomatic children attending a day-care centre in the State of São Paulo [75]. It should be noted that direct comparison of genotyping results and drawing of conclusions from these investigations should be made with caution because of the intrinsic differences in targeted population, sample size, sampling and diagnostic procedures, molecular tools, and marker (s) investigated.

In a previous molecular survey conducted in the State of Paraná, BIV (70.4%) and AII (22.2%) were the most prevalent *G. duodenalis* sub-assemblages found in individuals of all ages [51]. These authors also identified BIV in water samples intended for human consumption and irrigation, and in leave vegetables. Furthermore, the same research group reported in a subsequent study of the presence of AII and BIV in one and three, respectively, food handlers working in public schools in the very same state [66]. In the present survey, AII was more frequently detected than BIV (47.4 *vs* 28.9%). Remarkably, multiple sequence alignment analyses demonstrated that some of our BIV sequences were identical (e.g. KJ741322 *vs* MG807891) with those described by Colli et al. [51]. Taken together, these data strongly suggest that transmission of *G. duodenalis* in Paraná is primarily anthroponotic, either directly through contact with infected individuals or asymptomatic carriers or indirectly through ingestion of contaminated food/water. At present we do not have information regarding zoonotic transmission of giardiasis in Paraná. In this regard, domestic dogs have been proposed as suitable natural reservoirs of human infections in surveys conducted in other Brazilian regions [57, 69], although other studies only found host-specific *G. duodenalis* assemblages in the canine, feline, and cattle populations investigated [54]. Additionally, we observed here that AII was significantly more prevalent in females belonging to the age group 5–9 years, and that individuals infected by this *G. duodenalis* sub-assemblage were more likely to report gastrointestinal symptoms.

The molecular data presented here also confirm the high level of genetic diversity at the nucleotide level within assemblage B (but not assemblage A) sequences observed by our research group and others in different endemic areas globally [67, 68, 76–78]. Of interest is the identification of a genetic variant (MG807891) in a number of BIV sequences generated at *gdh* with a SNP (A to G) in position 180. The fact that this genotype seems rare or absent from Africa [67, 68] and Europe [79, 80] suggests that the presence of this SNP may be used as a molecular signature to predict the geographical origin of a given *G. duodenalis* sample. Overall, the considerable heterogeneity observed within our assemblage B sequences, together with the elevated rate of overlapping nucleotide peaks during chromatogram inspection and the lack of intra-assemblage mixed infections seem to support the occurrence of recombination events as the most likely explanation for this genetic variation within *Giardia* [81, 82].

This study is the first account describing the molecular diversity of *Blastocystis* in the State of Paraná and comprises the most extensive panel (*n* = 104) of *Blastocystis*-positive samples genotyped in Brazil to date. Our molecular analyses revealed the presence of six subtypes (ST1–4, ST6, and ST8) circulating in the surveyed general population, with ST1 and ST3 being the most prevalent (36.0 *vs* 40.4%) and ST2 ranking third (15.4%). Similar ST1/3 frequencies have been reported recently in poor communities [57] and paediatric [83] and clinical [84] populations in the State of São Paulo, and in patients (and their relatives) attending a day-care hospital for the mentally ill in Rio de Janeiro [85]. Additionally, ST2 has been shown as a relevant *Blastocystis* subtype in asymptomatic patients [86] and the Amazonian Tapirapé indigenous people [87]. Explicit sex and age-related distribution of *Blastocystis* subtypes were noticed in the population investigated, with ST3 carriage being significantly more detected in males younger than 10 years-old and ST1 carriage in females in the age group of 10–14 years. Of interest, 2.9% of the *Blastocystis* samples genotyped in the present study were assigned to ST4, a subtype commonly distributed in Europe but rare or absent in the Americas [15]. Because of its marked geographical distribution and primarily clonal structure, ST4 has been proposed as a lineage with a recent entry into the human population [88]. Indeed, in South America ST4 has been identified sporadically only in Colombian human and non-human primates [86, 89]. Remarkably, in Brazil ST4 has been reported so far in Paraná (present study) and previously in Rio de Janeiro [85, 90]. Because international travel has grown fast since 2000, it is reasonable to think that visiting tourists (mainly from Europe) are major contributors to the introduction and spreading of this specific *Blastocystis* subtype in Brazil. Furthermore, several molecular surveys have suggested a link between ST4 and the occurrence of gastrointestinal disorders. Thus, ST4 has been demonstrated as the most prevalent *Blastocystis* subtype identified in clinical specimens in Spain [91], in patients presenting with acute diarrhoea in Denmark [92] and patients suffering from irritable bowel syndrome and chronic diarrhoea in Italy [93]. In line with these data, in the present study, 66.7% (2/3) of ST4 carriers reported persistent diarrhoea and/or abdominal pain. Finally, ST6 and ST8 were detected in an insufficient number of samples. ST6 appeared to be common in birds and ST8 in some non-human primates [94, 95], but are rarely found in humans. These findings indicate that the origin of these ST6 and ST8 cases are very likely the result of zoonotic transmission events, as suggested by the large numbers of free-range poultry in the area and the proximity of wild populations of platyrrhine primates.

## Conclusions

In conclusion, and despite the apparent progress achieved in the control of EPs in the Paraná State in later years, data presented here demonstrate that protozoan and helminth parasites still represent a public health concern in the region. We also provide evidence of the usefulness of molecular-based methods, including PCR and sequence analyses, to ascertain the transmission dynamics of some of the investigated species, mainly *G. duodenalis* and *Blastocystis*, and the potential link between their genotype and pathogenicity. Finally, we believe that the epidemiological and molecular information generated here may help local and regional health authorities and decision makers to optimise available human and material resources when fighting against these infections.

## Additional files

Additional file 1: **Table S1.** Oligonucleotides used for the molecular identification and/or characterisation of *Giardia duodenalis* and *Blastocystis* sp. in this study. (DOCX 17 kb)

Additional file 2: **Table S2.** The frequency of single and multiple infections by enteric protozoan and helminth parasites over the total individuals infected by at least one enteric pathogen (*n* = 353) in Paranaguá, Paraná, Brazil, 2015–2016. (DOCX 28 kb)

## Abbreviations

*bg*: ß-giardin
CI: Confidence interval
C_q_: Cycle threshold
EP: Enteric parasites
GBD: Global burden of disease
*gdh*: Glutamate dehydrogenase
MRCT: Modified Ritchie concentration technique
NCBI: National Center for Biotechnology Information
PRR: Prevalence risk ratio
qPCR: Quantitative real-time polymerase chain reaction
SNP: Single-nucleotide polymorphism
*SSU* rRNA: Small subunit ribosomal RNA
ST: Subtype
STH: Soil-transmitted helminth
WHO: World Health Organization
