# Supplementary material for: Prevalence of intestinal parasites, with emphasis on the molecular epidemiology of Giardia duodenalis and Blastocystis sp., in the Paranaguá Bay, Brazil: a community survey
Source: Parasit Vectors. 2018 Aug 30;11:490. doi: 10.1186/s13071-018-3054-7 (PMC6117969; doi:10.1186/s13071-018-3054-7)
Supplement: Supplementary file 1 — Table S1. Oligonucleotides used for the molecular identification and/or characterisation of Giardia duodenalis and Blastocystis sp. in this study. (DOCX 17 kb) [file 13071_2018_3054_MOESM1_ESM.docx]

**Additional file 1: Table S1.** Oligonucleotides used for the molecular identification and/or characterization of *Giardia duodenalis* and *Blastocystis* sp. in this study.

| Target organism | Locus | Oligonucleotide | Sequence (5'–3') | Reference |
| --- | --- | --- | --- | --- |
| *Giardia duodenalis* | *ssu* rRNA | Probe | FAM–CCCGCGGCGGTCCCTGCTAG–BHQ1 | [37] |
|  |  | Gd-80F | GACGGCTCAGGACAACGGTT | [37] |
|  |  | Gd-127R | TTGCCAGCGGTGTCCG | [37] |
|  | *gdh* | GDHeF | TCAACGTYAAYCGYGGYTTCCGT | [38] |
|  |  | GDHiF | CAGTACACCTCYGCTCTCGG | [38] |
|  |  | GDHiR | GTTRTCCTTGCACATCTCC | [38] |
|  | *bg* | G7-F | AAGCCCGACGACCTCACCCGCAGTGC | [39] |
|  |  | G759-R | GAGGCCGCCCTGGATCTTCGAGACGAC | [39] |
|  |  | G99-F | GAACGAACGAGATCGAGGTCCG | [39] |
|  |  | G609-R | CTCGACGAGCTTCGTGTT | [39] |
| *Blastocystis* sp. | *ssu* rRNA | BhRDr | GAGCTTTTTAACTGCAACAACG | [40] |
|  |  | RD5 | ATCTGGTTGATCCTGCCAGT | [40] |
