# Supplementary material for: Prevalence of intestinal parasites, with emphasis on the molecular epidemiology of Giardia duodenalis and Blastocystis sp., in the Paranaguá Bay, Brazil: a community survey
Source: Parasit Vectors. 2018 Aug 30;11:490. doi: 10.1186/s13071-018-3054-7 (PMC6117969; doi:10.1186/s13071-018-3054-7)
Supplement: Supplementary file 2 — Table S2. The frequency of single and multiple infections by enteric protozoan and helminth parasites over the total individuals infected by at least one enteric pathogen (n = 353) in Paranaguá, Paraná, Brazil, 2015–2016. (DOCX 28 kb) [file 13071_2018_3054_MOESM2_ESM.docx]

**Additional file 2: Table S2.** The frequency of single and multiple infections by enteric protozoan and helminth parasites over the total individuals infected by at least one enteric pathogen (*n* = 353) in Paranaguá, Paraná, Brazil, 2015‒2016.

|  | **Cases (*n*)** | **Percentage (%)^a^** | **Percentage (%)^b^** |
| --- | --- | --- | --- |
| **Single infections** | 194 | 55.0 | 7.2 |
| *Giardia duodenalis* | 31 | 8.8 | 4.0 |
| *Blastocystis* sp. | 89 | 25.2 | 11.6 |
| *Endolimax nana* | 35 | 9.9 | 4.6 |
| *Iodamoeba butschlii* | 1 | 0.3 | 0.1 |
| *Entamoeba coli* | 5 | 1.4 | 0.7 |
| *Entamoeba* complex | 2 | 0.6 | 0.3 |
| *Entamoeba hartmanni* | 6 | 1.7 | 0.8 |
| *Chilomastix mesnilii* | 0 | 0 | 0 |
| *Retortamonas intestinalis* | 0 | 0 | 0 |
| *Ascaris lumbricoides* | 15 | 4.2 | 2.0 |
| *Trichuris trichiura* | 8 | 2.3 | 1.0 |
| Ancylostomatidae | 2 | 0.6 | 0.3 |
| *Strongyloides* spp. | 0 | 0 | 0 |
| *Enterobius vermicularis* | 0 | 0 | 0 |
| **Double infections** | 102 | 28.9 | 13.3 |
| *Giardia duodenalis* + *Blastocystis* sp*.* | 19 | 5.4 | 2.5 |
| *Giardia duodenalis* + *Endolimax nana* | 3 | 0.8 | 0.4 |
| *Giardia duodenalis* + *Entamoeba coli* | 1 | 0.3 | 0.1 |
| *Giardia duodenalis* + *Ascaris lumbricoides* | 2 | 0.6 | 0.3 |
| *Giardia duodenalis* + *Trichuris trichiura* | 5 | 1.4 | 0.7 |
| *Giardia duodenalis* + Ancylostomatidae spp. | 1 | 0.3 | 0.1 |
| *Blastocystis* sp*.* + *Endolimax nana* | 27 | 7.6 | 3.5 |
| *Blastocystis* sp*.* + *Iodamoeba butschlli* | 1 | 0.3 | 0.1 |
| *Blastocystis* sp*.* + *Entamoeba coli* | 8 | 2.3 | 1.0 |
| *Blastocystis* sp*.* + *Entamoeba hartmanni* | 5 | 1.4 | 0.7 |
| *Blastocystis* sp*.* + *Ascaris lumbricoides* | 7 | 2.0 | 0.9 |
| *Blastocystis* sp*.* + *Trichuris trichiura* | 4 | 1.1 | 0.5 |
| *Blastocystis* sp*.* + *Strongyloides* spp | 2 | 0.6 | 0.3 |
| *Blastocystis* sp*.* + *Enterobius vermicularis* | 2 | 0.6 | 0.3 |
| *Endolimax nana* + *Iodamoeba butschlii* | 2 | 0.6 | 0.3 |
| *Endolimax nana* + *Entamoeba coli* | 6 | 1.7 | 0.8 |
| *Endolimax nana* + *Entamoeba* complex | 1 | 0.3 | 0.1 |
| *Endolimax nana* + *Entamoeba hartmanni* | 2 | 0.6 | 0.3 |
| *Endolimax nana* + *Ascaris lumbricoides* | 1 | 0.3 | 0.1 |
| *Entamoeba coli* + *Ascaris lumbricoides* | 1 | 0.3 | 0.1 |
| *Entamoeba coli* + *Trichuris trichiura* | 1 | 0.3 | 0.1 |
| *Ascaris lumbricoides* + *Trichuris trichiura* | 1 | 0.3 | 0.1 |
| **Triple infections** | 42 | 11.9 | 5.5 |
| *Giardia duodenalis + Blastocystis* sp. + *Endolimax nana* | 6 | 1.7 | 0.8 |
| *Giardia duodenalis + Blastocystis* sp. + *Entamoeba* complex | 1 | 0.3 | 0.1 |
| *Giardia duodenalis + Blastocystis* sp. + *Ascaris lumbricoides* | 3 | 0.8 | 0.4 |
| *Giardia duodenalis + Blastocystis* sp. + *Trichuris trichiura* | 3 | 0.8 | 0.4 |
| *Giardia duodenalis + Endolimax nana* + *Trichuris trichiura* | 1 | 0.3 | 0.1 |
| *Giardia duodenalis + Entamoeba coli* + *Trichuris trichiura* | 1 | 0.3 | 0.1 |
| *Blastocystis* sp. + *Endolimax nana + Entamoeba coli* | 6 | 1.7 | 0.8 |
| *Blastocystis* sp. + *Endolimax nana + Entamoeba* complex | 1 | 0.3 | 0.1 |
| *Blastocystis* sp. + *Endolimax nana + Entamoeba hartmanni* | 7 | 2.0 | 0.9 |
| *Blastocystis* sp. + *Endolimax nana + Chilomastix mesnilii* | 1 | 0.3 | 0.1 |
| *Blastocystis* sp. + *Endolimax nana + Ascaris lumbricoides* | 2 | 0.6 | 0.3 |
| *Blastocystis* sp. + *Entamoeba complex + Entamoeba hartmanni* | 1 | 0.3 | 0.1 |
| *Blastocystis* sp. + *Entamoeba complex + Trichuris trichiura* | 2 | 0.6 | 0.3 |
| *Blastocystis* sp. + *Ascaris lumbricoides + Trichuris trichiura* | 3 | 0.8 | 0.4 |
| *Blastocystis* sp. + *Ascaris lumbricoides + Enterobius vermicularis* | 1 | 0.3 | 0.1 |
| *Blastocystis* sp. *+ Trichuris trichiura +* Ancylostomatidae | 2 | 0.6 | 0.3 |
| *Endolimax nana + Entamoeba* complex + *Trichuris trichiura* | 1 | 0.3 | 0.1 |
| **Quadruple infections** | 12 | 3.4 | 1.6 |
| *Giardia duodenalis + Blastocystis* sp. + *Endolimax nana + Entamoeba hartmani* | 3 | 0.8 | 0.4 |
| *Giardia duodenalis + Blastocystis* sp. + *Endolimax nana +* Ancylostomatidae spp. | 1 | 0.3 | 0.1 |
| *Giardia duodenalis + Blastocystis* sp. + *Entamoeba coli + Entamoeba hartmani* | 1 | 0.3 | 0.1 |
| *Blastocystis* sp. + *Endolimax nana + Iodamoeba butschlii + Entamoeba coli* | 1 | 0.3 | 0.1 |
| *Blastocystis* sp. + *Endolimax nana + Entamoeba* complex *+ Entamoeba hartmanni* | 1 | 0.3 | 0.1 |
| *Blastocystis* sp. + *Endolimax nana + Trichuris trichiura +* Ancylostomatidae | 1 | 0.3 | 0.1 |
| *Blastocystis* sp. + *Entamoeba coli + Entamoeba* complex *+ Ascaris lumbricoides* | 1 | 0.3 | 0.1 |
| *Blastocystis* sp. + *Retortamonas intestinalis + Trichuris trichiura +* Ancylostomatidae | 1 | 0.3 | 0.1 |
| *Endolimax nana + Entamoeba* complex *+ Entamoeba hartmanni + Chilomastix mesnilii* | 1 | 0.3 | 0.1 |
| *Endolimax nana + Entamoeba* complex *+ Entamoeba hartmanni + Retortamonas intestinalis* | 1 | 0.3 | 0.1 |
| **Quintuple infections** | 3 | 0.8 | 0.4 |
| *Giardia duodenalis + Blastocystis* sp. + *Endolimax nana + Entamoeba hartmani + Trichuris trichiura* | 1 | 0.3 | 0.1 |
| *Giardia duodenalis + Blastocystis* sp. + *Endolimax nana + Entamoeba* complex *+ Entamoeba hartmani* | 1 | 0.3 | 0.1 |
| *Blastocystis* sp. + *Endolimax nana + Entamoeba hartmanni + Chilomastix mesnilii + Ascaris lumbricoides* | 1 | 0.3 | 0.1 |

^a^ Over the total individuals infected by at least one enteric pathogen (*n* = 353).

^b^ Over the total individuals surveyed (*n* = 766).
